# Domestic

**Published:** 1841-07-03

**Authors:** 


### DOMESTIC.

### HEALTH OF THE CITY.

INTERMENTS *in the City and Liberties of Philadelphia, from the 19th of June, to the 26th.*

| Diseases. | Adults. | Children. |
|---|---|---|
| Abscess of Lungs, | 1 | 0 |
| Anorexia, | 1 | 0 |
| Apoplxy, | 3 | 0 |
| Casualties, | 1 | 1 |
| Consumption of the lungs, | 20 | 4 |
| Convulsions, | 0 | 7 |
| Diarrhœa, | 0 | 1 |
| Dropsy, | 0 | 1 |
| —— Abdominal, | 1 | 1 |
| —— Head, | 0 | 2 |
| —— Breast, | 1 | 0 |
| Disease of Heart, | 2 | 0 |
| —— Prostate, | 1 | 0 |
| —— Uterus, | 1 | 0 |
| Drowned, | 0 | 1 |
| Erysipelas, | 1 | 0 |
| Enlargement of heart, | 1 | 0 |
| Effects of Laudanum, | 0 | 1 |
| Fever, | 0 | 1 |
| —— Congestive, | 1 | 0 |
| —— Remittent, | 0 | 1 |
| —— Typhus, | 1 | 0 |
| —— Typhoid, | 2 | 0 |
| —— Puerperal, | 1 | 0 |
| —— Scarlet, | 0 | 1 |
| Gastrodynia, | 1 | 0 |
| Hæmorrhage from Uterus, | 1 | 0 |
| Inflammation of the Brain, | 1 | 0 |
| —— Bronchi, | 1 | 1 |
| —— Throat, | 0 | 2 |
| —— Stomach and Bowels, | 1 | 0 |
| —— Bowels, | 0 | 1 |
| | — | — |
| *Carried forward,* | 44 | 26 |
| *Brought forward,* | 44 | 26 |
| Inflammation of Heart, | 1 | 0 |
| —— Liver, | 1 | 1 |
| —— Kidneys, | 1 | 0 |
| Intemperance, | 0 | 1 |
| Marasmus, | 0 | 2 |
| Measles, | 0 | 2 |
| Mania a potu, | 3 | 0 |
| Old age, | 4 | 0 |
| Palsy, | 1 | 0 |
| Rupture of Aorta | 1 | 0 |
| Scirrhus of Uterus | 1 | 0 |
| Small pox, | 0 | 3 |
| Stone, | 1 | 0 |
| Still-born, | 0 | 11 |
| Summer Complaint, | 0 | 8 |
| Unknown, | 0 | 4 |
| | — | — |
| Total, 117— | 60 | 57 |

Of the above, there were under 1 year, 34

| | |
|---|---|
| From 1 to 2 | 5 |
| 2 to 5 | 6 |
| 5 to 10 | 8 |
| 10 to 15 | 1 |
| 15 to 20 | 3 |
| 20 to 30 | 13 |
| 30 to 40 | 13 |
| 40 to 50 | 10 |
| 50 to 60 | 8 |
| 60 to 70 | 8 |
| 70 to 80 | 6 |
| 80 to 90 | 2 |
| 90 to 100 | 0 |
| Total, | 117 |

Of the above there 14 were from the almshouse, and 17 people of colour, which are included in the total amount.

---

The last number of the Western Journal of Medicine and Surgery contains a case of ligature of the subclavian artery, by Professor Gross, together with a summary of the twenty-six cases in which this operation has been performed, with the results. The case of Dr. Gross we extract; its length prevents our referring at present to the remaining cases by other Surgeons, which thus associated present great interest.

*Case of Axillary Aneurism for which the Subclavian Artery was tied; with an inquiry into the nature and history of that affection.* By S. D. GROSS, M. D., Professor of Surgery in the Louisvillle Medical Institute.—Daniel Monday, a free negro, the father of several children, of a stout muscular frame, and temperate habits, 36 years of age, came under my care on Saturday, February 13th, 1841, on account of an aneurism of the right axillary artery. He was an industrious, hard-working man, a brickmaker by occupation, and had always enjoyed excellent health until within the last few months. On examination, I discovered a circumscribed, pulsating tumour underneath the pectoral muscle, extending from the clavicle, which it had thrown considerably upwards, into the axilla, on the one hand, and down towards the cartilage of the fourth rib on the other. It was of an irregular, conical shape, and about the size of a very large fist, measuring fully four inches at its base in one direction by three and a half in the other. In its feel it was very tense, as well as inelastic, and the pulsation was so distinct that it could be seen at the distance of some feet from the patient. Pressure, however firmly applied, produced no sensible effect upon it; and the blood rushed into it with a loud whizzing noise, or "bruit de soufflet." Owing to the elevation of the clavicle it was impossible to compress the subclavian artery so as to arrest the pulsation in the swelling, or even diminish the flow of blood through it. The whole limb, from the top of the shoulder to the extremities of the fingers, was benumbed, painful, and almost deprived of motion; the swelling, however, was slight, and not at all œdematous in its character: the temperature was also good. Severe suffering was likewise experienced in the site of the tumor and in the lower part of the neck, from the compression, doubtless, of the axillary plexus of nerves. The pectoral muscle was stretched to the greatest extent; and the patient constantly inclined his head towards the affected side, keeping the elbow nearly at a right angle, and supporting it carefully with the opposite hand, to prevent tension of the swelling. The pulse at the wrist was nearly as distinct as in the other limb, but it now and then intermitted. For the last four weeks the pain was almost incessant; it was particularly severe at the chest and shoulder, and had become so agonizing of late as to deprive him, in a great measure, of sleep, or even to prevent him from lying down. His appetite was also much impaired, and his countenance was expressive of the deepest distress.

On questioning him as to the origin of his disease, he stated that last Christmas a year ago, while firing a yager, the but-end struck against the right collar bone, near its middle, followed instantly by great numbness of the whole limb, which continued upwards of an hour, when it gradually subsided. Early in

April following, the arm began to swell from the wrist to the shoulder, attended with slight pain and impaired sensibility, which were supposed to be of a rheumatic character. The tumour was first perceived in June; it was situated just below the clavicle, pulsated very faintly, and did not exceed the volume of a hazel-nut. From this period on it gradually enlarged, having acquired by the following Christmas the dimensions of an apricot or moderate-sized plum. It had also become the seat of considerable pain; and there was a manifest increase of numbness in the affected side. In January the aneurism was observed to grow with great rapidity, the arm became gradually useless, and, in short, there was a decided aggravation of all the symptoms; the sufferings of the patient, when I first saw him, at the date above specified, being of the most excruciating nature.

As there was considerable vascular excitement with a coated condition of the tongue and constipation of the bowels, he was requested to be bled immediately to the amount of about twenty ounces, to take a liberal dose of senna and salts, to keep perfectly quiet, and to use none but the slightest kind of food. On Wednesday the 17th of February he called again at my room, when it was found that the tumour had much increased in size, being nearly or quite one-third larger than on the previous Saturday. The pain was also much greater; his appetite was extinct; and for the last three or four nights he had scarcely slept a wink. Under these circumsances, convinced that no time was to be lost, I immediately requested a consultation with my colleagues, Professors Drake, Cobb, and Miller. Dr. Bayles, our Demonstrator of Anatomy, Dr. T. L. Caldwell, Dr. Donne, and several other physicians were also present, and kindly offered their views concerning the case. After carefully deliberating upon the subject, these gentlemen unanimously concurred with me in the opinion that the only chance for the patient's life was to tie the subclavian artery; and the operation was accordingly appointed for twelve o'clock next day.

On Thursday, February 18th, in the presence of my colleagues, a large number of physicians, and of the medical class, I proceeded to the operation. The patient was placed upon a narrow table of moderate height, the head and chest were properly elevated with pillows, and the face turned a little towards the sound side, while an assistant pulled at the wrist, so as to depress, as much as possible, the affected shoulder. The integuments over the clavicle being next stretched upon the chest, I made my first incision along the centre of that bone, beginning near the sternal origin of the mastoid muscle and passing out towards the acromion process of the scapula for about three inches and a half; thus dividing at one stroke the common integuments and the fibres of the platysma-myoid. The parts being now allowed to retract, left the lower margin of the cut parallel, and on a level with the superior border of the clavicle. A second incision, about two inches in length, was carried along the posterior edge of the mastoid muscle, at a right angle with the preceding. The triangular flap thus formed was then dissected up and held out of the way, care being taken not to interfere with the external jugular vein, or any of the smaller arteries of the neck. Having advanced thus far, the cervical aponeurosis was detached from the clavicle by cautious strokes of the handle of the scalpel, which laid bare the brachial plexus of nerves and the omo-hyoid muscle. At this stage of the operation a small vein, a branch of the subclavian, was divided, and although it bled very little, it was immediately secured with a temporary ligature. Taking the omo-hyoid for my guide, I divided the loose cellular substance in the triangular space bounded above by the muscle just mentioned, by the clavicle below, and by the anterior scalene muscle internally, and thus approached the artery as it passed over the first rib. The vessel here lay at some distance from the inferior branch of the brachial plexus of nerves, rather deeply behind the collar-bone; and with a common aneurismal needle, armed with a double ligature of saddler's silk, no difficulty was experienced in securing it, the instrument being passed from before backwards and from below upwards. It was then drawn very firmly with the fingers and tied with a double knot within a few lines of the anterior scalene muscle: as soon as this was accomplished all pulsation in the sac, as well as at the wrist, ceased. One end of the ligature being cut off, the other was left protruding at the inner angle of the wound, the edges of which were closed by three sutures and adhesive straps. Not half an ounce of blood was lost during the operation, which lasted twenty minutes.

The man was put to bed, and the limb, laid in an easy position, wrapped in cotton wadding. In less than an hour, the temperature, which had been considerably depressed, was thoroughly restored; the pain and numbness had greatly abated; and the patient expressed himself more comfortable than he had been for a month.

8 o'clock, P. M.—He is quite comfortable, except a slight degree of soreness along the wound. His breathing is natural, and the pulse about 80. He was requested to lie perfectly quiet, to keep the limb well covered, and to drink toast-water.

19. 9 o'clock, A. M.—Had a pretty good night, having enjoyed several hours of sound and refreshing sleep. Tongue moist and tolerably clean: pulse 80: heat of the skin natural: no unusual thirst: temperature of the limb equal to that of the opposite side: *tumor quite solid.* Ordered thin gruel, as he expressed a wish to have something to eat.

8 o'clock, P. M.—Progresses very well; there being no fever, or any pain or tenderness in the wound: bowels have not been opened since the operation: is requested to take an ounce of sulphate of magnesia at 10 o'clock.

20. 9 o'clock, A. M.—Passed a good night: pulse soft, regular, and about 78 a minute: tongue moist and pretty clean: is free from pain, except at the back part of the shoulder and arm, where there is some degree of uneasiness: bowels relieved by the salts: numbness of the limb rapidly subsiding.

7 o'clock, P. M.—Has had another alvine evacuation: the wound exhibits a good appearance; and the patient is, in all respects, doing well.

21. 10 o'clock, A. M.—Removed the dressings, and cut away two of the sutures: the wound has united pretty well, except at each angle, where there is a discharge of healthy looking pus: parts beneath have a solid feel: tumor is considerably diminished in volume: patient rests well, and is entirely comfortable.

From this date every thing went on favorably. The tumor steadily decreased in size, the numbness of the limb gradually subsided, the hand and fingers could be more easily moved, the temperature was equal to that of the opposite arm, the patient had a good appetite, and, in short, every function appeared to be well executed. He was still kept on the lightest kind of food, and was forbidden even to raise himself up in bed: no medicine was administered, excepting occasionally a dose of oil or salts, to regulate the bowels. The other stitch was removed in a few days, and the wound rapidly cicatrized, excepting at the upper angle, and at the point where the ligature of the artery protruded.

The ligature came away on the morning of the fourteenth day from the operation, without a drop of blood. The patient at the time was in good health and spirits, and expressed a wish to return to his family, upwards of one hundred miles down the Ohio. The tumor had diminished fully one-half in volume, the pectoral muscle, beneath which it was situated, being quite flaccid and extensible. The pulse at the wrist was still absent, and there was likewise some numbness at the top of the shoulder and the extremities of the fingers, but the movements of the limb daily improved, and the patient was perfectly convalescent. To guard against accident, he was requested to live low, und keep his bed a few days longer.

On Sunday the 7th of March, being obliged to leave town, I placed the patient in the care of my friend Dr. T. L. Caldwell, to whom, as well as to Dr. S. B. Richardson, who was subsequently called in, I am greatly indebted both for his unweared attention and skilful treatment, and for an account of the daily progress of the case until my return. From the diary furnished by the former gentleman I make the following extracts.

Nothing worthy of notice occurred until Saturday night the 13th of March, when he began to feel somewhat unwell; he had walked about the room occasionally, and been out several times in the yard. Being no better next day, he took a dose of castor oil of his own accord, and on Monday morning an ounce of salts, both of which had operated well, and he expressed himself much relieved. He had experienced no pain, and attributed his indisposition to having taken cold from throwing off the bed-clothes on Sunday night. Dr. Caldwell, who saw him towards noon on Monday, found his pulse somewhat accelerated, with a slight degree of tenderness in the apex of the tumor, the volume of which had not perceptibly diminished since my departure. The wound was perfectly cicatrized, excepting at two points not exceeding a quarter of an inch in length, and from which there was a very small discharge of healthy matter. The pulse at the wrist was still imperceptible.

16. 5 o'clock, A.M.—About 12 o'clock last night the patient was suddenly seized with intense pain in the chest, which was particularly severe at the base of the right lung, from which it extended over towards the sternum, on the one hand, and up towards the axilla, on the other. Respiration short, hurried, laborious, and about 56 a minute: pulse 140, quick and tense: tongue moist and covered with a white coat: aneurismal tumor had suddenly disappeared last night at the time of the attack. Dr. Richardson having reached the patient a short time before Dr. Caldwell, they adopted the following treatment: bleeding at the left arm to about sixteen ounces, and the subjoined prescription, which was ordered to be repeated every two hours:

℞ Subm. Hydrarg. gr. iv.
Nitr. Potassæ gr. iii.
Antim. Tartariz. gr. $\frac{1}{4}$. M.

In addition to this, a solution of antimony, in the proportion of one grain to the ounce of water, was ordered to be given, in teaspoonful doses, at the alternate hour, to keep up slight nausea. The bleeding produced considerable relief of the pain, with a diminution of the tenseness and frequency of the pulse.

3 o'clock, P. M.—The pain, though somewhat abated, still persisting, he was cupped to six ounces at the lower part of the chest with a good deal of relief: had a small alvine dejection in the forenoon: suspend the mercurial powders, and continue the antimonial mixture.

6 o'clock, P. M.—Bronchial respiration throughout the right lung, with dulness on percussion over the lower ribs: the blood drawn this morning sizy and cupped. Ordered a large blister to the side, extending from the base of the chest to a short distance above the nipple: continue the antimony every hour.

17. 8 o'clock, A. M.—Blister has drawn well: there is some abatement of pain, the respiration is less frequent, and the pulse is also somewhat better: continue the antimonial mixture steadily, but give it in larger doses to produce nausea.

6 o'clock, P. M.—There is more vascular excitement: no motion from bowels since yesterday: twelve ounces of blood were taken from the arm, the pulse giving way under it: persevere with the previous medicine, and administer half an ounce of castor oil, repeating it every three hours, if necessary.

18. 8 o'clock, A. M.—Blood drawn last evening buffed: bowels not yet moved: the patient experiences a sensation near the superior part of the chest as if a fluid was passing from the pleuritic cavity into that of the aneurismal tumour, and on carefully auscultating the spot, Dr. Caldwell could distinctly hear a plashing sound at every inspiration, the noise resembling that produced by shaking water in a closed vessel: the respiration is still bronchial, though not so hurried and painful, and the dulness on percussion is perceived over a larger extent of surface than previously: the breathing is likewise of a bronchial character in the left lung: ordered one ounce of sulphate of magnesia, another blister to the upper part of the chest, and a continuance of the antimony.

6 o'clock, P. M.—Medicine has operated two or three times on the bowels: blister has drawn well: tongue coated and rather dry: *no pain in the chest:* breathing slightly improved.

19. 8 o'clock, A. M.—Had a tolerably good night: respiration still further improved: tongue dry throughout: pulse 140: continue the antimony.

2 o'clock, P. M.—Having returned to the city, I visited Daniel with Dr. Caldwell, Dr. Richardson, and Prof. Miller: pulse small and 140: breathing short, laborious, and 38 in a minute: extremities cold: surface bedewed with clammy perspiration: tongue dry and covered with a brownish-white coat: bronchial respiration in left lung, and almost complete absence of sound in the right: ordered

℞. Carb. Ammoniæ ʒii.

Pulv. G. Acaciæ ʒi.

Mist. Camph. ℥vi. M.

Give a large tablespoonful every hour, and drink milk punch freely.

9 o'clock, P. M.—Condition unchanged: continue as before.

20. 9 o'clock, A. M.—Appears to be somewhat better, the pulse having more volume and less frequency: dozed some during the night: has had five or six alvine evacuations since last evening: is very feeble, but still sensible: continue with the ammonia and milk punch, and take ten drops of laudunum every two hours until the diarrhœa is checked.

2 o'clock, P. M. Continues much the same, excepting that he is sensibly weaker. Towards evening the mind began to wander, deglutition became difficult, the pulse ceased in the left wrist, and about nine o'clock in the evening he expired apparently without pain, having survived the operation a little upwards of thirty days.

It is worthy of remark that the limb of the affected side retained its temperature fully up to the last moment, although the pulse at the wrist was at no time perceptible. It should also be observed that there was scarcely any cough, or expectoration, during the whole of the period which intervened between the rupture of the aneurismal sac and the patient's death.

*Autopsy.*—At my particular request the body was inspected, thirty-six hours after death, by Dr. T. L. Caldwell, Dr. Richardson, and Dr. Bayless, in the presence of a large number of the most respectable practitioners in the city, and several medical students. The preceding evening a cold injection of lead had been thrown into the innominata with such force as to pass into the brain and return by the left carotid and verbetral, at the same time filling pretty completely the arteries of the neck, the shoulder, and superior extremity of the affected side.

The body was somewhat emaciated; the wound made during the operation completely cicatrized; and the pectoral muscles considerably wasted, but in other respects unchanged. The subclavian artery terminated abruptly at the outer margin of the scalene muscle, where the ligature had been applied, *its caliber being closed by a mass of solid fibrin, about one-third of an inch in length, which adhered firmly to the lining membrane, and thus afforded an effectual barrier to the passage of the blood.* Between this and the thyroid axis the vessel was occupied by a dark coagulum of blood, which, as it was unadherent, was probably formed only a short time before death. Beyond the seat of the ligature, the artery had a rough, ragged appearance, and was sufficiently pervious to admit of the ready passage of a small probe into the aneurismal sac. Superiorly the tumour was overlapped by the brachial plexus of nerves, while in front, at its lower part, was the subclavian vein which, besides being thrown out of its natural course, was considerably diminished in size. No pus was any where discoverable, the parts immediately involved in the operation being intimately consolidated by plastic lymph. The aneurismal tumour, placed immediately below the clavicle, was of a conical form, and about the volume of a moderate-sized orange, being two inches and a quarter in diameter at its base. Its walls varied in thickness at different points from half a line to the eighth of an inch, and its interior communicated by means of an oval aperture, one inch and three quarters in length by an inch and a half in width, with the pleuritic cavity: it was situated between the first and second ribs, nearly equi-distant between the sternum and the spine, and was the result obviously of ulcerated absorption induced by the pressure of the tumour. Both ribs were denuded of their periosteum immediately around the opening, and the serous membrane had a shreddy, ragged aspect. The aneurismal sac contained a few reddish clots arranged in a laminated manner, and closely adherent to its inner surface, especially at the part corresponding with the apex of the tumour.

The right thoracic cavity contained nearly three quarts of bloody looking serum, intermixed with flakes of lymph and laminated clots, the latter of which were of a reddish-brown color, and had evidently been lodged originally in the aneurismal sac. The pleura exhibited everywhere marks of high inflammation, and the right lung was greatly reduced in volume, from the compression of the effused fluid. The left lung was considerably engorged, and at one or two points almost hepatized. The heart and pericardium were sound. The abdominal viscera presented nothing unusual. None of the arteries appeared to have been affected by disease.

The vessels which, by their communication with the subclavian artery above the ligature, and the axillary trunk below the tumor, were mainly instrumental in keeping up the circulation of the limb and shoulder, were the supra and infra scapular, together with the short thoracic. The former of these branches arose on the tracheal side of the scalene muscle, and passing over its interior surface, about three quarters of an inch above the clavicle, soon separated into three twigs, one of which ran towards the root of the coracoid process, the other two towards the posterior part of the scapula. The trunk of the vessel did not appear to be particularly enlarged, but its terminal divisions were nearly twice the ordinary volume. The infra-scapular artery, which arose from, and consequently opened into, the lower part of the sac, was nearly as capacious as the axillary, or parent trunk. It soon divided into two large branches, one of which formed the long thoracic artery, while the other was distributed to the subscapular muscle. The short thoracic vessels presented nothing worthy of note.

In reflecting upon the manner in which this case terminated, the question naturally arises, did the ulcerative absorption which gave rise to the opening above referred to, and which finally led to the escape of a portion of the contents of the aneurismal sac, commence prior to the deligation of the subclavian artery on the 18th of February, or not until some time subsequently? For my own part, I am disposed to believe that it was set up fairly before the operation was performed; a view of the case which is fully borne out, I think, by several circumstances, such especially as the extraordinary bulk of the tumor, and the violent pulsation that was constantly going on in its interior. Under the influence of these two causes, ulcerative absorption must have commenced, in all probability, several months—certainly six or seven weeks—before the occurrence of the perforation which ultimately destroyed the patient. As soon as the operation was over all pulsation in the tumor ceased; its contents became speedily solidified; and its volume progressively diminished, so that for upwards of three weeks there was every reason to believe the patient would rapidly recover. Indeed, no case could have presented more flattering prospects. After the artery was tied the pressure occasioned by the swelling must have been comparatively slight, as was evinced by the fact that the pain and numbness, which before had been constant and excessive, almost immediately subsided. Hence it follows that ulcerative absorption would be much more likely to have been induced before than after the operation. The denuded condition of the ribs, the ragged state of the neighbouring pleura, and the absence of purulent matter in the sac and in the wound, the latter of which was perfectly healed when the man died, all concur, moreover, to induce the belief that the perforating process had been going on for some time prior to the performance of the operation.

Another question of no little interest in referrence to the case before us, may be asked here, namely, what would have been the probable effect of opening the aneurismal sac and clearing it of coagulated blood, soon after the artery was tied? Could the event which occurred have been foreseen, such a procedure, I conceive, would not only have been warrantable, but highly improper; but under the circumstances it would not have been right, certainly not in accordance with the general experience of the profession on this point, to attempt it. The practice, once so much in vogue, of laying open the sac must, to say the least of it, be a very dangerous one; that it was almost universally so previously to the time of John Hunter is too well known to require any comment on this occasion, and that it would be less so at the present day, even with our improved methods of operating, admits, I presume, of much doubt.

Be this as it may, the case, considered in relation to the manner in which it terminated, may be regarded as altogether unique. The only instance, so far as my information extends, which is at all similar to it, is one narrated by Mr. Guthrie, in his work on the diseases and injuries of the arteries. As it is highly important, both in a pathological and practical point of view, no apology need be offered for introducing a brief outline of it in this place.

A stout, middle-sized man, 53 years of age, became a patient in one of the London hospitals, on account of an aneurismal swelling immediately below the right clavicle, which was first noticed in November, soon after lifting a heavy weight. It gradually increased in size until the following August, when, unwilling to submit to an operation, he went into the country. In December he returned in great suffering, and requested the assistance of Mr. Guthrie. He now experienced continued pain and numbness in the shoulder, chest, and arm of the affected side, with a teazing cough and purulent expectoration; the tumour, which pulsated strongly and with a peculiar thrill, was of the volume of a forty-eight pound shot; and the clavicle was pushed high up into the neck. In a few days after this the patient began to spit blood, which gradually increased in quantity until his death, which happened in three weeks from the time of his return to London. On opening the body it was found that the aneurism had forced its way into the right thoracic cavity by the removal of a part of the first five ribs, had contracted adhesions to the superior lobe of the lung, and had finally opened into it, so that the gradual discharge of blood destroyed the patient.

